# Disruption of the Aldehyde Dehydrogenase 2 Gene Results in No Increase in Trabecular Bone Mass Due to Skeletal Loading in Association with Impaired Cell Cycle Regulation Through p21 Expression in the Bone Marrow Cells of Mice

**DOI:** 10.1007/s00223-017-0285-0

**Published:** 2017-05-04

**Authors:** Kayoko Furukawa Okuma, Kunitaka Menuki, Manabu Tsukamoto, Takafumi Tajima, Hokuto Fukuda, Yasuaki Okada, Toshiharu Mori, Takuto Tsuchiya, Toshihiro Kawamoto, Yasuhiro Yoshida, Soshi Uchida, Akinori Sakai

**Affiliations:** 1 Department of Orthopaedic Surgery, School of Medicine, University of Occupational and Environmental Health, 1-1 Iseigaoka Yahatanishi-ku, Kitakyushu, 807-8555 Japan; 2 Department of Environmental Health, School of Medicine, University of Occupational and Environmental Health, Kitakyushu, Japan; 3 Department of Immunology and Parasitology, School of Medicine, University of Occupational and Environmental Health, Kitakyushu, Japan; 4 Department of Orthopaedic Surgery, Wakamatsu Hospital for the University of Occupational and Environmental Health, Kitakyushu, Japan

**Keywords:** Aldehyde dehydrogenase 2, Mechanical loading, p21, Cell cycle

## Abstract

Approximately 45% of people of East Asian descent have the inactive aldehyde dehydrogenase 2 (ALDH2) phenotype. The enzyme defect of ALDH2 has been found to adversely influence the risk of osteoporosis. The aim of this study was to clarify the effect of skeletal loading on trabecular bone structure and dynamics in Aldh2-disrupted mice in the absence of alcohol consumption. Four-week-old male Aldh2^−/−^ (KO) and Aldh2^+/+^ (WT) mice were divided into a ground control (GC) group and a climbing exercise (CE) group in each genotype. The trabecular bone mineral density of the distal femur measured by peripheral quantitative computed tomography in the wild-type CE (WTCE) group was significantly higher than that in the wild-type GC (WTGC) group; however, there was no significant difference between the knockout CE (KOCE) and knockout GC (KOGC) groups. Bone histomorphometry revealed that osteogenic parameters were significantly increased in the WTCE group compared with the WTGC group, but not increased in the KOCE group compared with the KOGC group. Quantitative reverse transcriptase polymerase chain reaction and flow cytometry revealed that mRNA and protein expression levels of p21 were significantly decreased in the WTCE group compared with those in the WTGC group, while these differences were not observed between the KOGC and KOCE groups. This study provides the first in vivo evidence that p21 expression in the bone marrow is not decreased after skeletal loading and osteoblast differentiation is impaired in the absence of Aldh2 gene.

## Introduction

The point mutation in aldehyde dehydrogenase 2 (ALDH2) that is identified as ALDH2*2 is the most frequent variant in humans and is present in 8% of the world’s population, including approximately 560 million people of East Asian descent. ALDH2*2 has an identifiable clinical phenotype that involves facial flushing and an increased heart rate when alcohol is consumed [1, 2].

The current consensus is that the ALDH2*2 variant is a risk factor for Alzheimer’s disease, Parkinson’s disease, cardiac ischemia, stroke, and osteoporosis [2]. An initial case–control study in Japan suggested that people with an ALDH2*1/*2 or ALDH2*2/*2 genotype had a greater tendency to develop late-onset Alzheimer’s disease [3]. ALDH1A1 and ALDH2 double-knockout (KO) mice exhibit a Parkinson-like phenotype, suggesting the importance of ALDH enzymes in protecting neurons in the substantia nigra from cellular damage [4]. A genome-wide association study in Japanese men also found that the ALDH2*2 variant increased the risk of coronary artery disease and myocardial infarction [5, 6]. This genetic polymorphism (ALDH2*2) is linked to more severe outcomes from ischemic heart damage and an increased risk of cell cycle arrest. ALDH2*2 is also involved in carcinogen metabolism, alcohol metabolism, and cell cycle control with a risk of head and neck cancer [7, 8]. ALDH2 is known to play a role in the regulation of cell cycle, although the precise mechanism is not well understood. Among a cohort of Japanese participants, a genetic screen identified ALDH2*2 as being associated with an increased osteoporosis risk [9]. However, how the ALDH2 gene is associated with osteoblastic differentiation and trabecular bone mass remains unclear.

In our previous study, we found that alcohol intake resulted in less trabecular bone volume (BV/TV) in Aldh2 KO (Aldh2^−/−^) mice compared with that in WT (Aldh2^+/+^) mice. The reduced formation of bone in the alcohol-induced bone loss was associated with elevated expression level of p21, a modulation of cell cycle progression in the bone marrow cells of the Aldh2^−/−^ mice [10]. We previously developed a mouse model of voluntary climbing exercise (CE) and reported that the CE increased bone formation in Aldh2^+/+^ mice [11]. However, the effect of the Aldh2 gene on the skeletal phenotype remains unknown. The aim of this study was to clarify the effect of skeletal loading on trabecular bone structure and dynamics in Aldh2-disrupted mice in the absence of alcohol consumption. We hypothesized that a disruption of the Aldh2 gene would result in no or less increase in trabecular bone mass in association with impaired cell cycle regulation after skeletal loading.

## Materials and Methods

### Experimental Animals

Male C57BL/6J (Aldh2^+/+^) mice, at 4 weeks of age, were purchased from Charles River Japan (Tokyo, Japan) and male Aldh2^−/−^ mice, at 4 weeks of age, were generated as described previously [12, 13]. The Aldh2^−/−^ mice were backcrossed with a C57BL/6J strain for more than 10 generations. All the mice were housed under standard laboratory conditions (temperature 24 ± 1 °C, humidity 55 ± 5%). The light/dark cycle was 12 h with lights on from 7:00 a.m. to 7:00 p.m. Drinking water and food were available ad libitum. The amount of food was matched among the experimental groups and enough not to cause starvation. All the mice were fed a commercial mouse diet (CE-2; Japan CLEA, Tokyo, Japan) containing 1.25% calcium and 1.06% phosphorus. The body weight was measured weekly. The experimental protocol was approved by the Ethics Review Committee for Animal Experimentation of the University of Occupational and Environmental Health.

### Experimental Design

Skeletal loading was achieved using the voluntary CE model described previously [11]. The mice without the voluntary CE were caged under the same conditions and pair fed with mice with voluntary CE. The mice in the ground control (GC) group lived under normal loading conditions, and the mice in the voluntary CE group lived in a climbing tower cage. The mice in the Aldh2^+/+^ (WT) group and Aldh2^−/−^ (KO) group were divided into two groups each: the WT mice in the GC (WTGC) group, the WT mice in the voluntary CE (WTCE) group, the KO mice in the GC (KOGC) group, and the KO mice in the voluntary CE (KOCE) group. The total experimental period in each group was 28 days. Bone labeling with a subcutaneous injection of calcein (20 mg/kg body weight) was performed at 7 and 3 days before death in the mice used for histomorphometry. At the end of the experiments, the mice were sacrificed by exsanguination under ether anesthesia. A total of 140 mice (WT and KO), at 4 weeks of age, were assigned to the two groups in each according to a stratified continuous randomization based on body weight. Twenty out of 140 mice were used for dual-energy X-ray absorptiometry (DXA), peripheral quantitative computed tomography (pQCT), microCT, and bone histomorphometric analysis. Forty mice were subjected to bone marrow cell culture. Forty mice were used for flow cytometric analyses and quantitative real-time reverse transcriptase polymerase chain reaction (RT-PCR). The remaining 40 mice were used for cell cycle analysis.

### Climbing Exercise

The CE mice were housed in cages containing a steel wire mesh tower consisting of cross stripes at the intervals of approximately 5 mm, as described previously [11, 14]. The mice voluntarily climbed up the 100-cm tower to drink water from bottles placed at the top of the cage. The mice were monitored every other day per week for 24 h/day during the experimental period using a CCD video camera (IVIS HF M52; Canon, Tokyo, Japan). The daily distances of climbing activity and daily climbing time period were calculated from the monitoring records. We also checked the amount of drinking water during the observation period of 4 weeks.

### Body Weight and Bone Sizes

The body weight of the mice was measured on days 0, 7, 14, 21, and 28. The right femurs were prepared by removing all the soft tissue and measured with a digital caliper (Digimatic Caliper; TopMan, Hyogo, Japan). The length of the femurs represented the distance between the top of the greater trochanter to the distal end of the lateral femoral condyle [15, 16].

### Examination of Bone Mineral Density

We measured bone mineral density (BMD, mg/cm^3^) on day 28 in the right femur using DXA (DCS-600, Aloka, Tokyo, Japan). We used five mice per group. Moreover, the right femurs were scanned using pQCT (XCT Research SA+, Stratec Medizintechnik GmbH, Pforzheim, Germany). The slices were located 1.2 mm from the growth plate (the distal metaphyseal site) in the femur. The trabecular region was determined in contour mode 2 and peel mode 2 with a threshold value of 395 mg/cm^3^. The trabecular BMD (mg/cm^3^) was measured at the distal metaphysis of the right femur. We also scanned the distal end of the femurs using a CosmoScan GX 3D microCT (Rigaku, Tokyo, Japan). MicroCT images were obtained using the following parameters: 90 kV, 88 μA, FOV 10 mm, voxel size 20 μm × 20 μm × 20 μm, and scan time 4 min.

### Bone Histomorphometry

The left proximal tibial specimens were embedded in methyl methacrylate acid after Villanueva’s bone staining on day 28. We used five mice per group. Non-decalcified 5-μm-thick coronal sections were cut with a microtome (model 2050 Supercut, Reichert-Jung, Heidelberg, Germany). Goldner staining was performed on the slices of Villanueva bone staining to make bone structure more obvious. Histomorphometry was performed using a semiautomatic image analysis system linked to a light microscope (Histometry-RT, System Supply, Nagano, Japan). The secondary spongiosa area was determined for each section. To exclude the primary spongiosa, the regions within 250 μm of the growth plate and one cortical shell width of the endocortical surface were not measured [17].

Regarding the structural parameters of the proximal tibia, the trabecular bone volume/tissue volume (BV/TV, %) was measured, and the mineral apposition rate (MAR, μm/day) and the surface referent bone formation rate (BFR/BS, μm %/day) were obtained using luminescence microscopy as reported previously [18].

We measured the osteoblast number/bone surface (Ob.N/BS, #/mm). We identified the cuboidal cells on the trabecular surface as osteoblasts. Trabecular thickness (Tb.Th, μm) was calculated by parallel plate model assuming constant geometry [11]. For the bone resorption parameter, the ratio of osteoclast number to bone surface (Oc.N/BS, #/mm) was obtained. We identified the cells that formed resorption lacunae on the trabecular surface and contained two or more nuclei as osteoclasts. We identified the surface of the lacuna generated by osteoclasts as eroded surface (ES/BS, %) [10, 14]. The abbreviations for the histomorphometric parameters were derived from the recommendations of the American Society of the Bone and Mineral Research Histomorphometry Nomenclature Committee [18].

### Cell Cultures

#### Preparation of Bone Marrow Cells

Bone marrow from the bilateral femurs and tibias was flushed out from the metaphysis with 5 ml of α-minimum essential media (α-MEM, Nakalai Tesk, Kyoto, Japan) on days 4 and 7. We used five mice per group. To assay alkaline phosphatase-positive (ALP^+^) colony forming unit fibroblast (CFU-f) colony formation, the marrow cells were plated at 1 × 10^6^ cells/well in six-well plates (Iwaki, Tokyo, Japan) in α-MEM containing 10% fetal bovine serum (Biowest, Dominican Republic), 2.0 g/l NaHCO_3_, 100 μg/ml streptomycin, 100 U/ml penicillin, 0.25 μg/ml amphotericin B, and 50 μg/ml ascorbic acid (Wako Pure Chemicals, Osaka, Japan). The cells were cultured at 37 °C in a humidified atmosphere of 5% CO_2_ in air with medium, which was changed every other day.

#### ALP^+^ CFU-f

On the 10th experimental day, CFU-f colonies were fixed with fixation solution and treated with a substrate tablet for ALP (BCIP-NBT solution kit for ALP stain, nuclease tested; Nacalai Tesque, Kyoto, Japan) dissolved in ddw at 37 °C. Colonies comprising 50 cells were defined as CFU-f. We counted the total and ALP^+^ CFU-f colonies with the culture dishes backlit at a fivefold magnification [16, 19–25].

### RNA Isolation and First-Strand cDNA Synthesis

Bone marrow cells from the bilateral femurs of mice were flushed out from the distal end of the metaphysis with 5 ml of phosphate-buffered saline (PBS) on day 4. We used five mice per group. Total RNA was extracted using an acid guanidinium thiocyanate–phenol–chloroform method and cleaned using the RNeasy kit from Qiagen (Hilden, Germany). The RNA was quantified spectrophotometrically, and agarose gel electrophoresis was then used to test the integrity of the RNA preparation. First-strand cDNA was reverse transcribed from total RNA (1 μg) using Moloney murine leukemia virus reverse transcriptase (SuperScriptII; Life Technologies, Rockville, MD, USA) and oligo(dT) 12–18 primers (Life Technologies).

### Quantitative RT-PCR

The quantitative RT-PCR analysis was performed using an iCycler apparatus (Bio-Rad Laboratories, Hercules, CA) with iCycler optical system software version 3.1 (Bio-Rad). Quantitative PCR reactions for p21, p53, cyclin-dependent kinase 1 (CDK1), and β-actin were performed in 20 μl with 7.5 ng of cDNA, 0.5 pM primers, and 10 μl of iQ SYBR Green Supermix (Bio-Rad). The PCR products of mouse p21 (470 bp), p53 (517 bp), CDK1 (128 bp), and β-actin (646 bp) cDNAs were obtained by amplification using primers for *p21* (5′-CTTTGACTTCGTCACGGAGAC-3′ and 5′-AGGCAGCGTATATACAGGAGAC-3′), *p53* (5′-CTTAGAGTTAAAGGATGCCCATGCTAC-3′ and 5′-AGAGGGAGACAGGGTGGGGGGTGGATA-3′), *CDK1* (5′-AGAAGGTACTTACGGTGTGGT-3′ and 5′-GAGAGATTTCCCGAATTGCAGT-3′), and *β*-*actin* (5′-CCTAAGGCCAACCGTGAAAAG-3′ and 5′-TCTTCATGGTGCTAGGAGCCA-3′). The primers used in this study were designed using Primer3 software and synthesized at Sigma-Aldrich Japan K.K. Genosys Division (Hokkaido, Japan). β-Actin served as an internal control. The amplification conditions were an initial 3 min at 95 °C and 40–50 cycles of denaturation at 95 °C for 30 s, annealing at each temperature for 30 s, and extension at 72 °C for 30 s. The mRNA expression levels were normalized to β-actin mRNA expression and expressed as relative values (fold change) compared to the expression levels in the WTGC group [10, 14, 25–27].

### Cell Cycle Analysis in Bone Marrow Cells

#### Flow Cytometry

Four groups consisting of five mice each were subjected to the same procedures as described above up to and including the harvesting of bilateral tibias on days 4 and 7. Bone marrow cells were obtained from both tibias. After washing with PBS, the samples were suspended in PBS containing 0.1% Triton X-100 and 0.5% ribonuclease A (Sigma). Cellar DNA was labeled with 50 μg/ml propidium iodide (Sigma). The cell cycle phase distribution in the stained cells was analyzed using a flow cytometer (EC800, Sony Biotechnology, Inc., Tokyo, Japan) [10, 28].

#### Membrane Preparation and Immunofluorescence Flow Cytometry

We used five mice per group. The bone marrow cells flushed from both tibias on days 4 and 7 were cultured under the standard culture conditions in six-well culture plates overnight. The adherent cells were scraped and centrifuged, washed twice with PBS, and incubated with 1 ml of FCM permeabilization buffer (Santa Cruz Biotechnology, Inc.) for 15 min at 4 °C. The cells were then washed with PBS and stained for 30 min on ice with anti-p21 primary antibody (sc-397, Santa Cruz Biotechnology, Inc.), followed by washing twice with PBS and staining with anti-rabbit IgG-FITC (fluorescein isothiocyanate) (sc-2012, Santa Cruz Biotechnology, Inc.) for 30 min at 4 °C; the cells were then washed with PBS. The stained cells were analyzed using a flow cytometer (EC800, Sony Biotechnology, Inc., Tokyo, Japan) to measure the percentage of p21-positive cells among the adherent cells [10].

### Statistical Analysis

We observed that the BV/TV value was 11.9 ± 1.2 (mean ± SD) after 28-day CE and that in the GC it was 7.6 ± 1.2 (mean ± SD) in WT mice based on our previous experiments [14] before starting the present study. To determine the number of mice required for meaningful statistical analysis, we performed power analysis before the conduct of the study. We used 3% in BV/TV as the minimum difference we wished to detect as significant and a standard deviation of 1.2%, assuming *α* = 0.05 (two-sided) and *β* = 0.2 (power = 80%). The analysis identified four as the minimum number of subjects required for each group. Actually, post hoc test identified that the experiment of five mice used in each group for BV/TV revealed that the *β* value was less than 0.2 (power is more than 80%).

All values for the results were expressed as the mean ± standard error (SE). A two-way factorial analysis of variance with Bonferroni was used to detect the potential effects of the gene KO and CE. Statistically significant differences were determined at *p* < 0.05. Calculations were performed using the IBM SPSS Statistics version 21 software (IBM Japan, Tokyo, Japan) on a Macintosh computer.

## Results

### Body Weight and Femoral Bone Length

There were no significant differences in body weight among the groups on day 0 (data not shown). The body weight (mean ± SE) on day 28 was 23.2 ± 0.3, 24.5 ± 0.4, 22.4 ± 0.4, and 21.4 ± 0.8 g in the WTGC, WTCE, KOGC, and KOCE groups, respectively. The body weights of the WTCE mice were significantly higher than those in the other groups. There were no significant differences in the length of the right femur among the groups on day 28 (data not shown).

### Climbing Distance and Time Period

The daily climbing distances (m/day) in the WTCE group after 1, 2, 3, and 4 weeks were 60.7 ± 2.1, 61.5 ± 0.6, 73.0 ± 1.7, and 65.6 ± 0.5, respectively. Those in the KOCE group after 1, 2, 3, and 4 weeks were 57.3 ± 1.1, 61.5 ± 1.3, 71.8 ± 1.5, and 66.5 ± 1.0, respectively. No significant differences were found among these values. No significant differences were also found in daily climbing time period and in the amount of drinking water among the four groups of WTGC, WTCE, KOGC, and KOCE during the observation period of 4 weeks.

### Bone Mineral Density

**Fig. 1:**
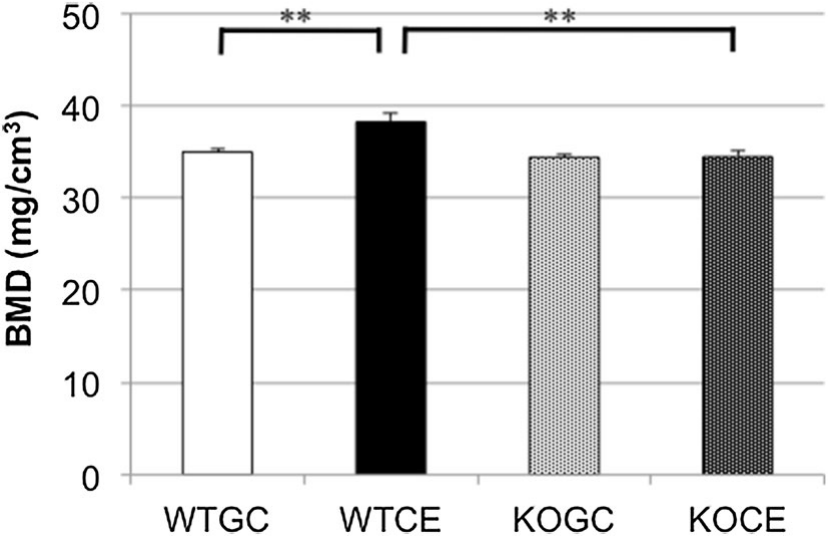
Trabecular BMD evaluated by DXA in whole femurs. The data are shown as the mean ± SE (*n* = 5 in each group). **p < 0.01. *BMD* bone mineral density

**Fig. 2:**
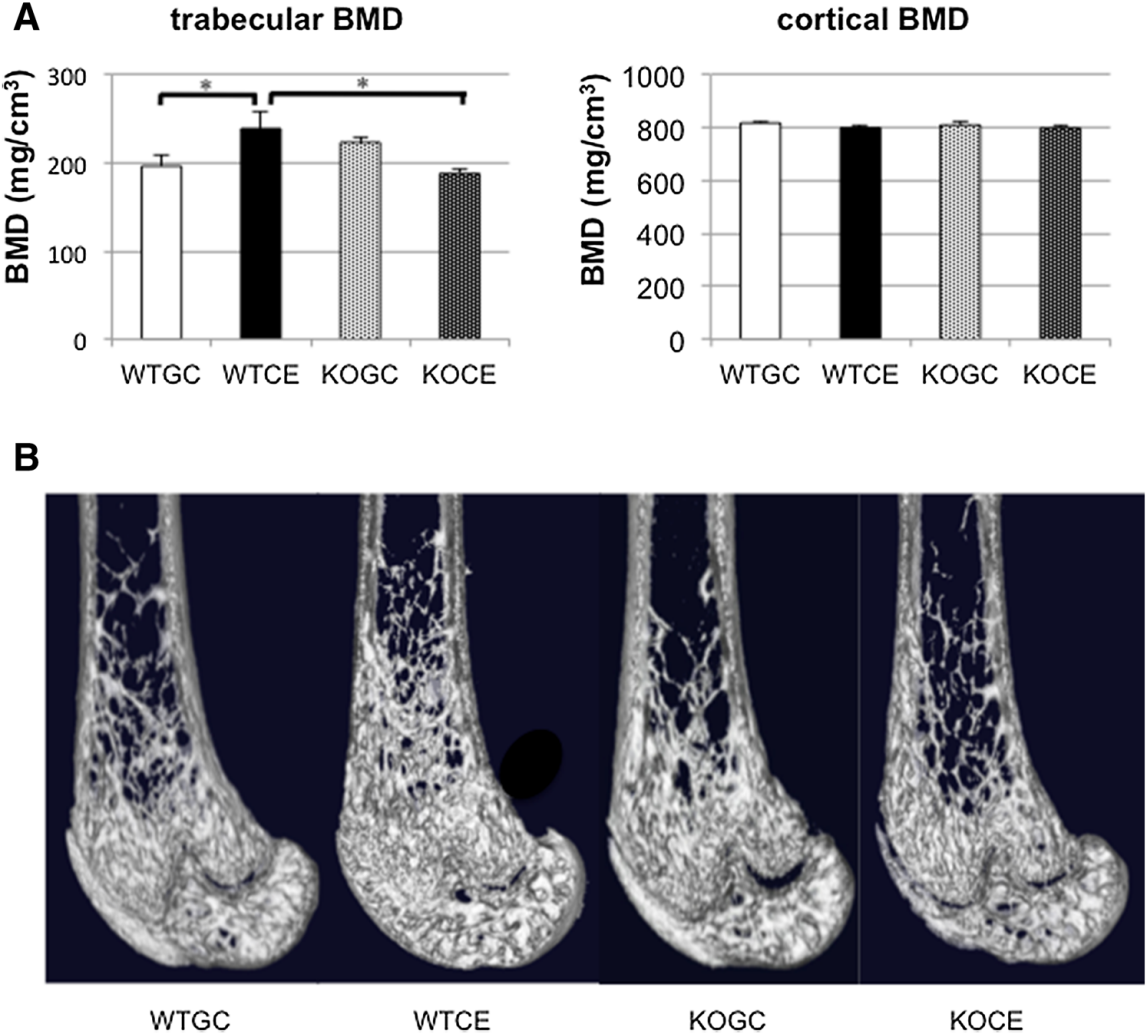
**a** Trabecular and cortical BMD evaluated by pQCT in the distal femurs. The data are shown as the mean ± SE (*n* = 5 in each group). *p < 0.05. *BMD* bone mineral density. **b** MicroCT images on day 28

The BMD of the right femur measured using DXA in the WTCE group on day 28 was significantly higher than that in the WTGC group, but there was no significant difference between the KOGC and KOCE groups (Fig. 1). The trabecular BMD (mg/cm^3^) of the distal femur measured using pQCT in the WTCE group (238.6 ± 18.7) was also significantly higher than that in the WTGC group (196.1 ± 12.8). However, there was no significant difference between the KOGC (223.1 ± 6.4) and KOCE groups (187.6 ± 5.5; Fig. 2a). There were no significant differences in cortical BMD among the four groups of WTGC (816.6 ± 6.6), WTCE (798.0 ± 7.8), KOGC (808.4 ± 16.1), and KOCE (796.9 ± 14.0). The microCT images also showed the increased trabecular bone of the distal femur in the WTCE group compared with those in the other three groups (Fig. 2b).

### Bone Histomorphometry

#### Trabecular Bone Volume and Structure of the Proximal Tibia

**Fig. 3:**
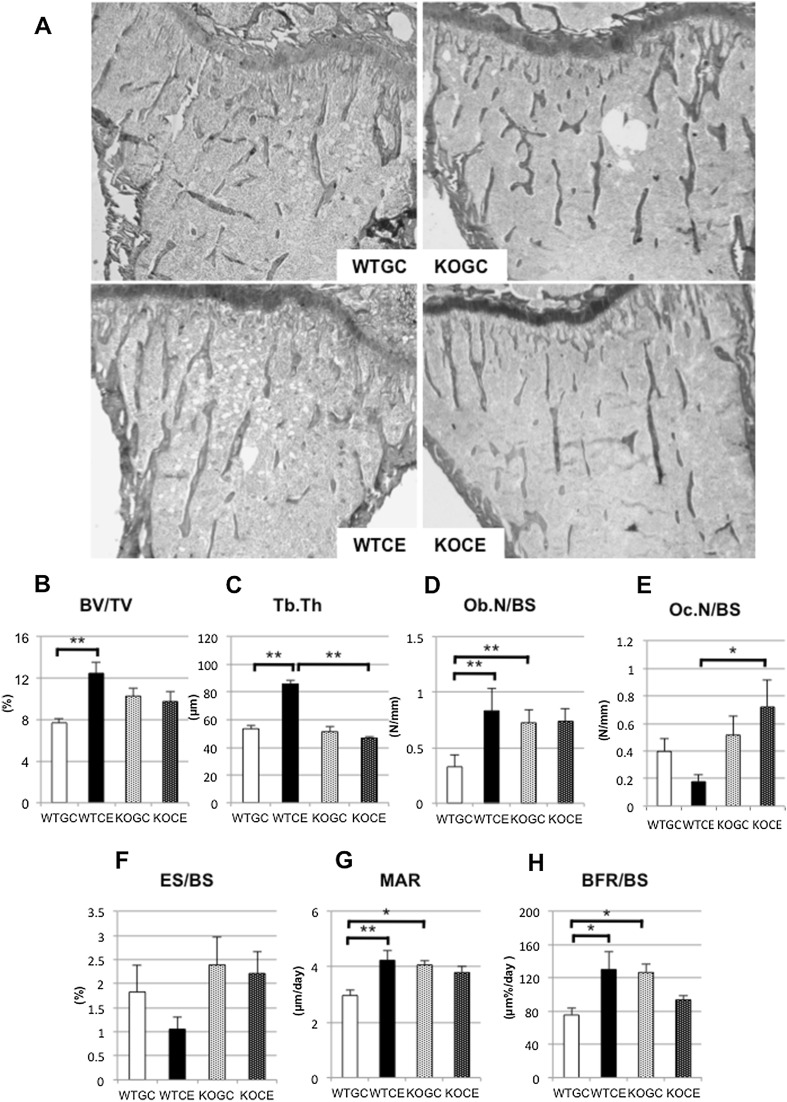
**a** Microscopic appearance of trabecular bone in the proximal tibias stained with Villanueva Goldner using the non-decalcified specimens (magnification, ×100). **b** BV/TV, **c** Tb.Th, **d** Ob.N/BS, **e** Oc.N/BS, **f** ES/BS, **g** MAR, and **h** BFR/BS. The data are shown as the mean ± SE (*n* = 5 in each group). *p < 0.05, **p < 0.01

The microscopic appearance of the proximal tibia showed that trabecular BV/TV in the area of the secondary spongiosa was increased in the WTCE group compared to that in the WTGC group, but that in the KOCE group was not increased compared with that in the KOGC group (Fig. 3a). On day 28, the value of trabecular BV/TV (%) at the proximal tibia in the WTCE (12.5 ± 1.1) group was significantly higher than that in the WTGC group (7.7 ± 0.4; Fig. 3b). There was no significant difference between the KOGC (10.3 ± 0.8) and KOCE (9.7 ± 0.9) groups. The value of Tb.Th (μm) at the proximal tibia in the WTCE group (85.8 ± 3.0) was also significantly higher than that in the WTGC group (53.4 ± 2.8; Fig. 3c). There was no significant difference between the KOGC (51.3 ± 3.9) and KOCE (46.7 ± 1.0) groups.

#### Trabecular Osteoblast Parameter

On day 28, the value of Ob.N/BS (#/mm) in the WTCE group (0.83 ± 0.21) was significantly more increased than that in the WTGC group (0.33 ± 0.11). However, there was no significant difference between the KOGC (0.72 ± 0.11) and KOCE (0.74 ± 0.12) groups (Fig. 3d).

#### Trabecular Osteoclast Parameter

On day 28, the value of Oc.N/BS (#/mm) in the KOCE group (0.72 ± 0.19) was significantly greater than that in the WTCE group (0.18 ± 0.05). There was no significant difference between the GC and CE mice in either the WT or KO group (Fig. 3e). There were no significant differences in the values of ES/BS among the four groups (Fig. 3f).

#### Dynamic Parameters of Trabecular Bone Formation

On day 28, the value of MAR (μm/day) was significantly higher in the WTCE group (4.2 ± 0.4) compared to that in the WTGC group (3.0 ± 0.2). There was no significant difference between the KOGC (4.1 ± 0.2) and KOCE (3.8 ± 0.2) groups (Fig. 3g). The BFR/BS value (μm %/day) was also significantly higher in the WTCE group (130.0 ± 21.9) compared to that in the WTGC group (75.4 ± 8.5). There was no significant difference between the KOGC (126.5 ± 10.1) and KOCE (93.5 ± 4.9) groups (Fig. 3h).

### Cell Cultures

To assay ALP^+^ CFU-f formation, primary cell culture was performed using bone marrow cells flushed out from the bilateral tibias and femurs on days 4 and 7.

#### ALP^+^ CFU-f

**Fig. 4:**
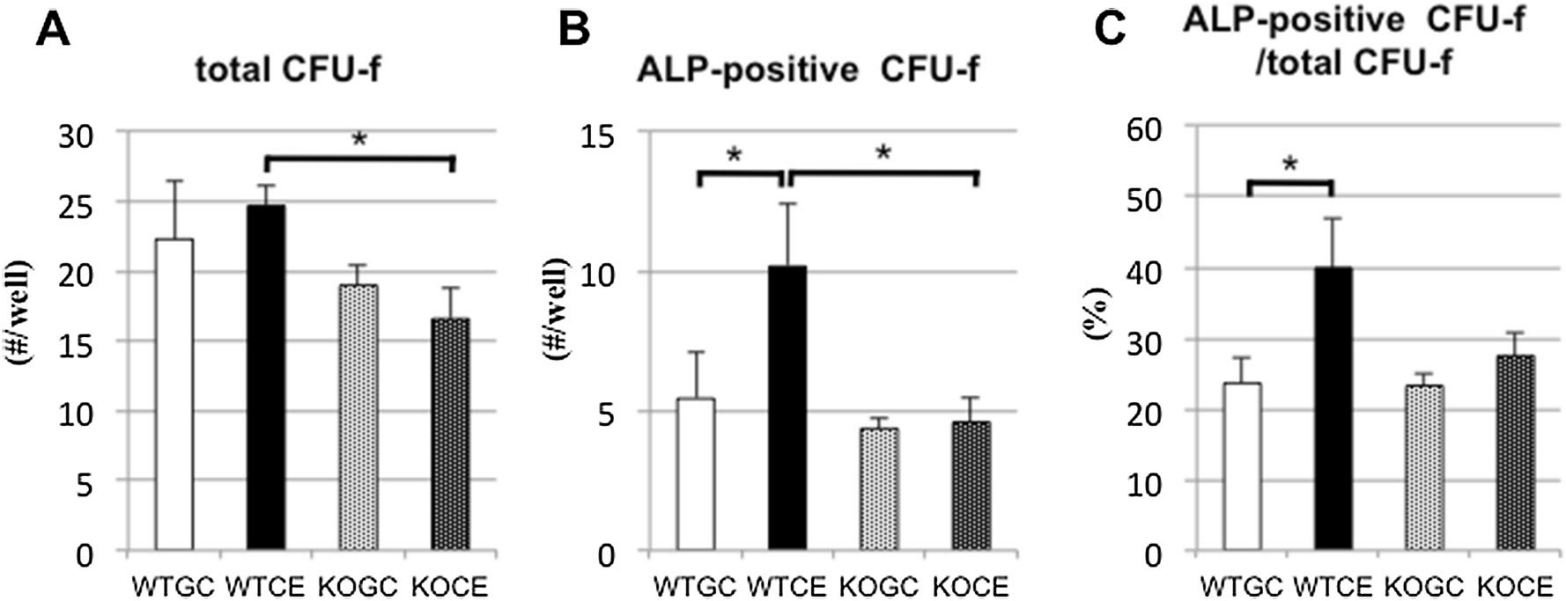
Osteogenic potentials evaluated by cell culture assay using bone marrow cells flushed from the bilateral femurs and tibias on day 7: **a** number of total CFU-f colonies per well, **b** number of ALP^+^ CFU-f colonies, and **c** percentage of ALP^+^/total CFU-f colonies. The data are shown as the mean ± SE (*n* = 5 in each group). *p < 0.05

Using cells flushed from the bilateral tibias and femurs, there were no significant differences in the total CFU-f numbers between the GC and CE groups in either the WT or KO mice (Fig. 4a). There was a significant difference in ALP^+^ CFU-f numbers between the WTGC and WTCE groups, but not between the KOGC and KOCE groups on day 7 (Fig. 4b). There was no significant difference in the ALP^+^ CFU-f numbers on day 4 among the mice in the four groups (data not shown). There was a significant difference in the ALP^+^ CFU-f/total CFU-f percentage between the WTGC and WTCE groups, but not between the KOGC and KOCE groups on day 7 (Fig. 4c).

### Expressions of p21, p53, and CDK1

**Fig. 5:**
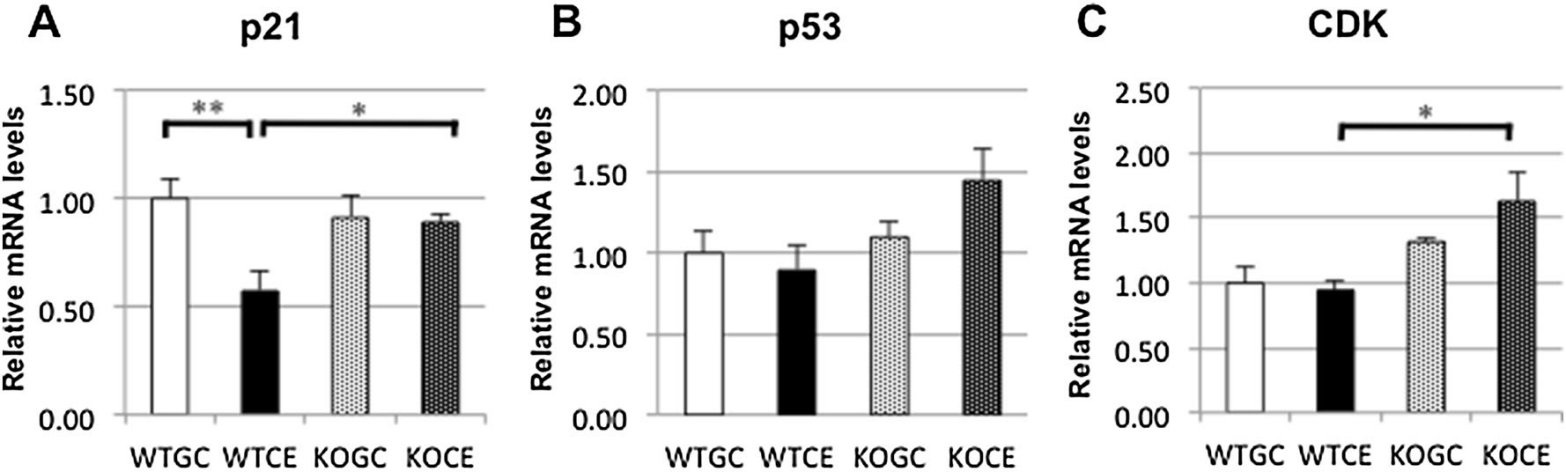
mRNA expressions in bone marrow cells measured by quantitative RT-PCR assays on day 4: **a** p21, **b** p53, and **c** CDK. The data are shown as the mean ± SE (*n* = 5 in each group). *p < 0.05, **p < 0.01

On day 4, the p21 mRNA expression level in the WTCE group was significantly lower than that in WTGC group (Fig. 5a). There were no differences in the mRNA expression levels of p53 and CDK1 between the GC and CE groups in either the WT or KO mice (Fig. 5b, c). On day 7, there were no differences in the mRNA expression levels of p21, p53, and CDK1 among the four groups (data not shown).

#### p21-Positive Cells in Adherent Bone Marrow Cells

**Fig. 6:**
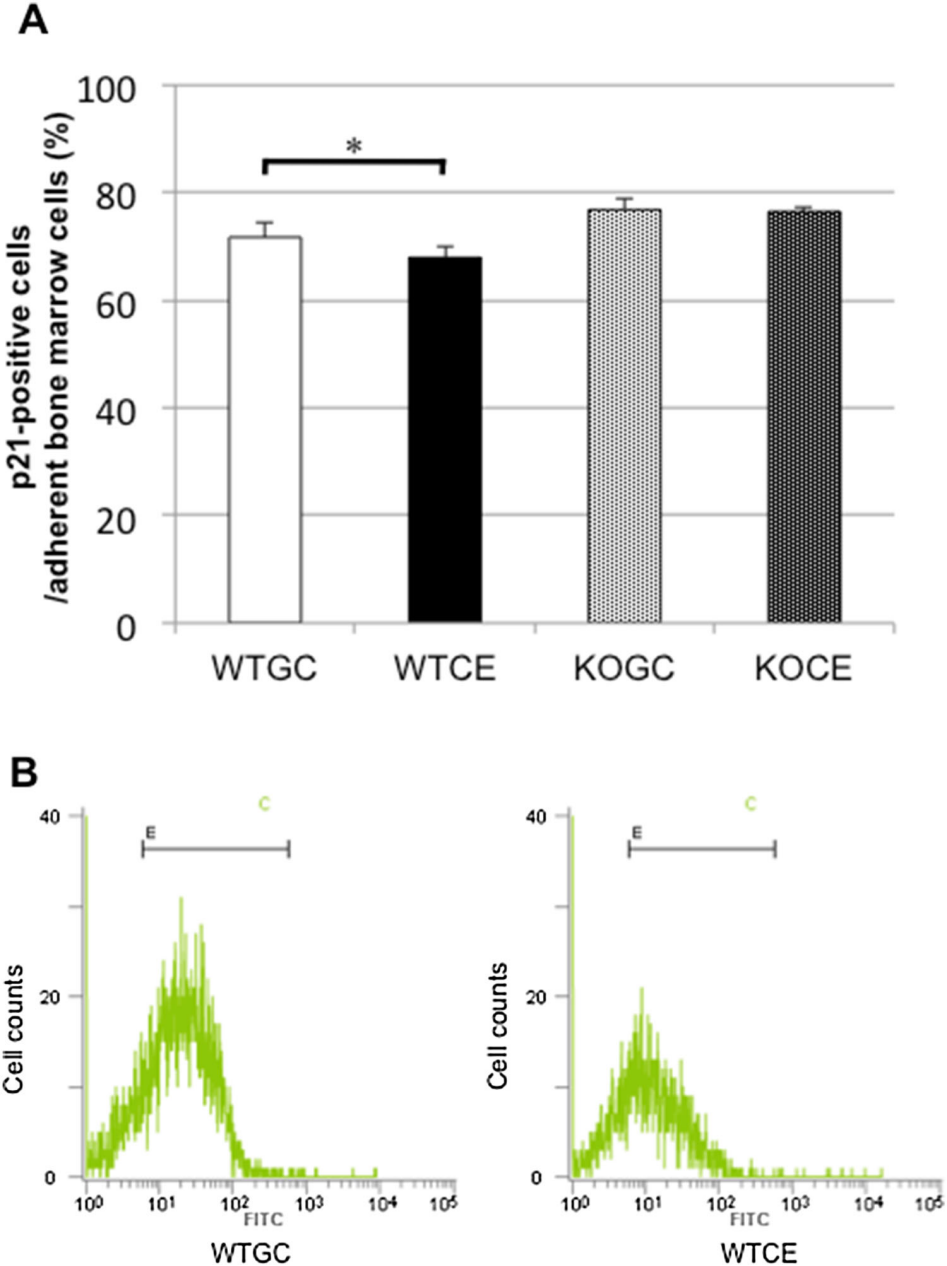
p21-positive cells. **a** Flow cytometric analysis of p21-positive cells in bone marrow adherent cells on day 7. **b** Histograms of the cell number in the WTGC and WTCE groups. The *x*-axis represents FITC intensity/cell, and the *y*-axis represents the number of cells registered/channel. The data are shown as the mean ± SE (*n* = 5 in each group). *p < 0.05

Although there were no significant differences in p21 antigen expression levels in the total population of bone marrow cells among the four groups on day 4 (data not shown), the expression level of p21 antigen in the WTCE group was significantly lower than that in the WTGC group on day 7 (Fig. 6a, b). The expression of p21 antigen in the KOCE did not differ from that in the KOGC group.

#### Cell Cycle Analysis of Bone Marrow Cells

**Fig. 7:**
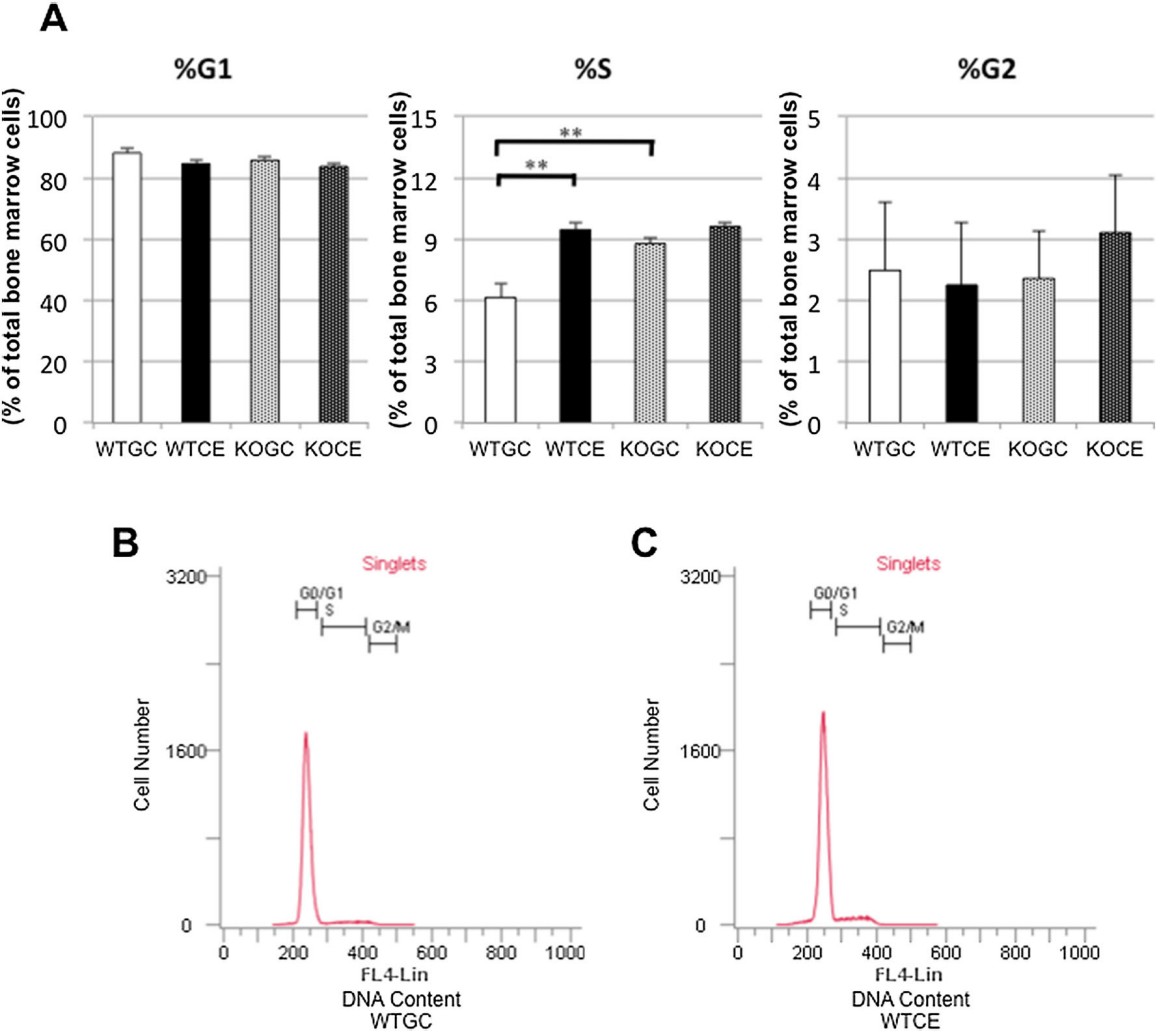
Cell cycle phase distribution of DNA in bone marrow cells. **a** Percentage of bone marrow cells in the G1, S, and G2 phases on day 4. Histograms of the cell number in the **b** WTGC and **c** WTCE groups. The *x*-axis represents the DNA content and the *y*-axis represents the number of cells. The data are shown as the mean ± SE (*n* = 5 in each group). **p < 0.01

The percentage of bone marrow cells in the S phase in the WTCE group was significantly higher than that in the WTGC group on day 4, while that in the KOCE group did not differ from that in the KOGC group. There was no significant difference in the percentage of bone marrow cells in the G1 and G2 phases among the four groups (Fig. 7a–c). There were no significant differences among the four groups in any phase on day 7 (data not shown).

## Discussion

Our study clearly demonstrated that the disruption of the aldehyde dehydrogenase 2 gene resulted in a lack of increase in trabecular bone mass after CE in association with no decrease in p21 expression in the bone marrow after skeletal loading. Whole femoral BMD was significantly increased after 28 days of CE in the WT mice but not in the KO mice. We confirmed the same bone characteristics using pQCT, which can be used to analyze trabecular and cortical BMD separately. There was a significant difference in the trabecular BMD between the WTGC and WTCE groups but not between the KOGC and KOCE groups. The histomorphometric study also revealed that trabecular BV/TV in the WTCE group was significantly increased compared with that in the WTGC group, but that in the KOCE group was not increased compared with that in the KOGC group. Ob.N/BS, Tb.Th, MAR, and BFR/BS in the WTCE group were significantly increased compared with those in the WTGC group. However, no significant differences were found in those parameters between the KOGC and KOCE groups. The CE promoted bone formation in the WT mice but not in the KO mice. The KOGC group consistently showed higher values of Ob.N/BS, MAR, and BFR/BS compared with the WTGC group. We have already observed higher osteogenic activities in osteoblasts in Aldh2^−/−^ mice [29]. However, BMD and bone formation parameters of bone histomorphometry in the KOCE group were not increased after CE. It should be especially mentioned that CE was not effective on trabecular bone increase in Aldh2^−/−^ mice in spite of high osteogenic activities. The cell culture study showed that colony formation was adequate in all the groups, but the osteogenic potential of the bone marrow cells was significantly promoted in the WTCE group compared with the other three groups.

To investigate the reason why bone formation in the KO mice was not promoted by the CE, we focused on the expression levels of osteogenic mRNAs and key cell cycle molecules based on our previous reports [10, 25, 27]. We detected a decrease in p21 mRNA expression in the bone marrow cells using quantitative RT-PCR after CE. These results were consistent with the results of flow cytometric analysis using bone marrow adherent cells that revealed a significant decrease in p21 protein expression in the WTCE group compared with that in the WTGC group. These differences were not observed between the KOGC and KOCE groups. Thus, we consider that the down-regulation of p21 is the key for osteogenesis in trabecular bone after CE.

Yew et al. reported that the knockdown of p21^Cip1/Waf1^ enhances cell proliferation, the expression of stemness markers, and osteogenic potential in human mesenchymal stem cells. These authors also reported that p21-shRNAs promoted cell proliferation and increased the percentage of cells in the S phase of the cell cycle compared with a control shRNA [30]. Blaber et al. reported that the microgravity of spaceflight induces pelvic bone loss through osteoclastic activity, osteocytic osteolysis, and osteoblastic cell cycle inhibition by CDKN1a/p21 [31]. This report is the first to posit a relationship between mechanical loading and p21 expression. Bellosta et al. determined that p21 acts as a brake in osteoblast differentiation [29]. The role of p21 is that of a cell cycle regulatory gene [32–35]. Our data indicating no increase in trabecular bone mass with a lack of p21 reduction after skeletal loading in the KO mice are comparable to those of previous reports. Shirakawa et al. found that mechanical stress tended to enhance the levels of the S/G2/M-phase cell fraction of the cell cycle [36]. Thus, the S phase of the cell cycle can be enhanced by either the absence of p21 expression or mechanical stress. In our study, the percentage of cells in the S phase of the cell cycle was significantly increased after the CE in the WT mice. The decreased p21 expression after the voluntary CE could regulate the cell cycle and result in increased trabecular bone formation.

We have three limitations in this study. The first is that the body weight of mice in the WTCE group was significantly higher than those in the other three groups despite pair feeding. Body weight is one of the confounding factors for bone mass. We guess that increased body weight in the WTCE group contains not only trabecular bone but also muscles during the growing period. Further investigation is needed for the alteration of body composition in WT and KO after CE. The second limitation is that the mechanism underlying how ALDH2 regulates p21 expression after CE without alcohol intake remains unclear. We measured enzyme activities of ALDH2 between the WTGC and WTCE groups with blood serum samples using ELISA kit (LS-F19070), Life Span Bioscience (WA). There was no significant difference between the two groups on days 4 and 7 (data not shown). The mRNA expression of Aldh2 in WTCE group did not differ from that in WTGC group (data not shown). Thus, we should further study cellular metabolic turnover as well as signal transduction of Aldh2. The third limitation is that the cultured bone marrow cells are crude and contain various types of adherent cells. However, in our previous study [10], the percentage of p21-positive cells was increased in adherent bone marrow cells after alcohol intake in Aldh2 KO mice, while the WR-PAK18, a p21-activated kinase-specific inhibitor, significantly prevented the reduction of mineralized nodule formation and the expression level of osteocalcin mRNA of adherent bone marrow cells reduced after alcohol intake in Aldh2 KO mice. The inhibition of the elevated p21 expression of adherent bone marrow cells resulted in the prevention of the reduction of mineralized nodule formation and expression level of osteocalcin mRNA. Thus, we think that p21 expression of adherent bone marrow cells is closely associated with osteogenic activity.

In our study, a time lag was observed among the phenomena of mRNA second protein expressions in bone marrow cells, protein expression of bone marrow adherent cells, and trabecular bone formation. On day 4 after CE, the mRNA and protein expression levels of p21 were significantly decreased, and the percentage in the S phase was significantly higher in the WTCE group than that in the WTGC group in the bone marrow cells. On day 7, the protein expression of p21 in the bone marrow adherent cells of the WTCE group was significantly lower than that in the WTGC group. The protein expression of p21 in bone marrow adherent cells is down-regulated after p21 mRNA expression declines. On day 28, the phenomenon of an increase in trabecular bone was finally observed.

This report is the first regarding the relationship between trabecular bone and mechanical loading in the presence or absence of Aldh2 gene, and we also suggest that p21 plays an important role in the pathway for the stimulation of osteoblastogenesis by mechanical loading. The increase in trabecular bone mass is associated with Aldh2 gene through the regulation of the cell cycle by a reduction in p21 levels after mechanical loading. In conclusion, in the absence of Aldh2 gene, mechanical loading did not increase trabecular bone mass in association with a lack in the decreased expression of p21.
